# Success rates and safety of a modified percutaneous PD catheter placement technique: Ultrasound-guided percutaneous placement of peritoneal dialysis catheters using a multifunctional bladder paracentesis trocar

**DOI:** 10.1097/MD.0000000000029694

**Published:** 2022-08-05

**Authors:** Zhen Li, Zheng Fang, HongYun Ding, JiYe Sun, Yi Li, Jie Liu, YunLu Yu, JianBin Zhang

**Affiliations:** a Department of Nephrology, The people’s hospital of Banan District, ChongQing, China; b Department of Radiology, The people’s hospital of Banan District, ChongQing, China; c Department of Nephrology, YongChuan Hospital of ChongQing medical university, ChongQing, China.

**Keywords:** multifunctional bladder paracentesis trocar, peritoneal dialysis catheters, percutaneous, safety, success rates, ultrasound-guided

## Abstract

**Background:**

We modified the blind Seldinger technique by incorporating ultrasound guidance and the use of a multifunctional bladder paracentesis trocar for PD catheter (PDC) placement, which can be easily performed by a nephrologist and is a feasible technique. To compare success rates and safety of our modified percutaneous PD catheter placement technique to open surgery.

**Methods:**

Two hundred and twelve stage-5 chronic kidney disease(CKD) patients receiving PD therapy from June 2016 to June 2019 were included, 105 patients treated by ultrasound-guided percutaneous placement of peritoneal dialysis catheters using a multifunctional bladder paracentesis trocar (Group A) and 107 patients receiving open surgical placement (Group B). Outcomes of patients via either catheter placement technique were retrospectively compared. The clinical success rate as defined by proper catheter drainage within 4 weeks after placement, complication rates (both technical complications and infections), and 1-year catheter survival were compared.

**Results:**

There was no significant difference in sex ratio, age, or previous abdominal surgery history between groups (*P* > .05). Both surgical time and incision length were significantly shorter in Group A than in Group B (*P* < .05). Clinical success rate was also higher inGroup A (*P* < .05). Moreover, Group A demonstrated lower overall complication rates (*P* < .05) and lower incidence rates of early peritonitis, initial drainage disorder, and peritubular leakage (all *P* < .05). One-year catheter survival was also higher in Group A (*P* < .05).

**Conclusion:**

Percutaneous placement of PD catheters using our modified technique demonstrates superior success rates and safety compared to open surgery. In addition, our modified technique can be a better alternative to traditional Seldinger percutaneous catheterization for its higher success rate and safety, more accurate positioning.

## 1. Introduction

At present, the most commonly used methods for peritoneal dialysis catheters (PDCs) placement are open surgery, laparoscopy, and percutaneous placement based on the Seldinger technique. Open surgical placement is the most widely used method which must be completed by experienced surgeons because it is traumatic to patients and technically difficult.^[1,2]^ Laparoscopy catheterization requires expensive equipment and professionally trained personnel not always available in newly established or small PD centers.^[3,4]^ Alternatively, percutaneous catheterization is a less invasive bedside technique mainly implemented by nephrologists, which is simple, clinically effective, and relatively inexpensive as confirmed by many clinical studies.^[5]^ However, as this placement technique is blind and limited by the surgical incision, it is impossible to look directly into the pelvic cavity, and difficult to accurately place the dialysis catheter in the appropriate position based on feel.^[6]^ It is also unsuitable for obese patients or patients with a history of abdominal surgery, thus hindering the extensive development of this technology.

To improve the technical reliability and safety of PDC placement, we modified the blind Seldinger technique by incorporating ultrasound guidance and the use of a multifunctional cystostomy paracentesis trocar for percutaneous puncture. This new technique can be easily performed by a nephrologist and is a feasible technique for ESRD patients.^[7]^ In the current study, we compared the clinical efficacy and safety of the new technique to open surgical placement.

## 2. Data and Methods

### 2.1. Study subjects

From June 2016 to June 2019, 228 stage-5 chronic kidney disease (CKD) patients requiring renal replacement therapy at the PD center of Yongchuan Hospital of Chongqing Medical University were considered for inclusion, with 16 patients eventually excluded (6 dropped out of the study and 10 were lost to follow-up), remaining 212 patients were included in the study. All patients provided written informed consent and the study protocol was approved by the institutional research ethics committee. The cohort included 105 patients treated by our modified percutaneous PD catheter placement technique (Group A) and 107 patients receiving open surgical placement (Group B). Baseline clinicodemographic characteristics are summarized in Table 1.

**Table 1:** Patient characteristics.

		A	B	*P* value
Patients	N	105	107	–
Male	N	60	61	.36
Age (y)	Mean±SD	49.35 ± 14.32	52.39 ± 15.18	0.57
Primary disease [n (%)]				
Glomerulonephritis		60 (58.3%)	50 (46.73%)	.94
Hypertensive nephropathy		10 (9.7%)	22 (20.56%)	.64
Diabetic nephropathy		21 (20.40%)	28 (26.17%)	.87
Others		12 (11.6%)	9 (8.41%)	.83
Previous abdominal surgery		5 (4.80%)	9 (8.41%)	.54

**Figure 1.**
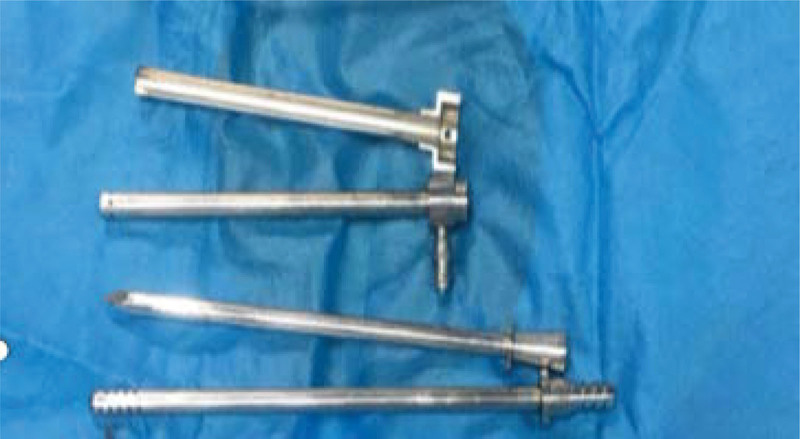
Details of the multifunctional bladder paracentesis trocar. The multifunctional cystostomy paracentesis trocar was an 18F stainless steel kit consisting of a semi-ring outer sheath (A), an inner trocar sheath (B), a sharp-headed trocar core (C), a blunt-headed trocar core (D).

The primary endpoint of this study was a functional catheter 1-month postinsertion (defined as technical success). Potential early complications considered for analysis were bowel perforation, hemorrhage in the rectus muscle or pelvic cavity, peritonitis, pericatheter leakage, and poor drainage. The secondary endpoint was 1-year technical survival, which was analyzed separately concerning insertion technique. The PD catheters used consisted of a double-cuff Tenckhoff straight tube (Baxter, USA) with an overall length of 41 cm and a diameter of 0.5 cm, in which the first polyester sheath was 16 cm from the catheter tip (Quinton Instrument Company, Seattle, WA, USA). The multifunctional cystostomy paracentesis trocar was an 18F stainless steel kit produced by Donghai Medical Devices (Zibo, Shandong Province, China) consisting of a semi-ring outer sheath, an inner trocar sheath, a sharp-headed trocar core, and a blunt-headed trocar core (Figure 1), which is originally designed to bladder ostomy for patients with blocked urethra and inability to urinate normally.

### 2.2. Procedure details

#### 2.2.1. Modified percutaneous placement of the PDC.

**Figure 2.**
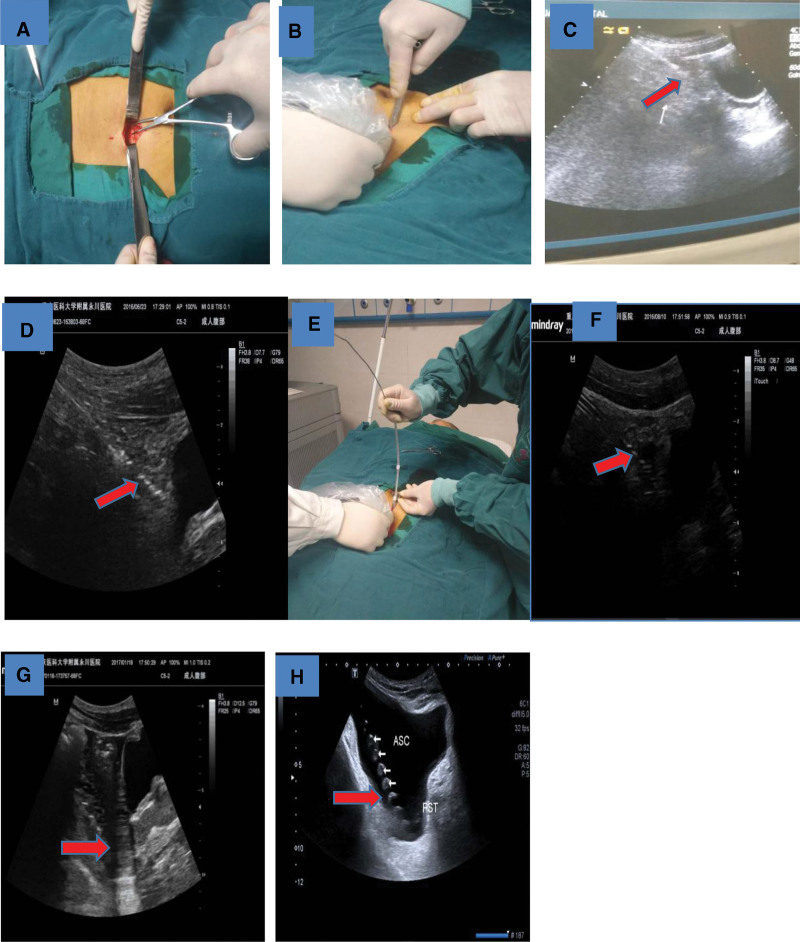
Procedure details of modified percutaneous placement of the PDC.

The procedure was performed by a nephrologist referring to our modified technique which we reported earlier,^[7]^ incorporating ultrasound guidance and the use of a multifunctional cystostomy paracentesis trocar for percutaneous puncture. The multifunctional cystostomy paracentesis trocar component has integrated functions of sharp-headed trocar core puncture, blunt-headed trocar core guidance, and semiring outer sheath blunt dilation by pulling out the built-in trocar core (Fig. 1). Detailed operation steps were shown in Fig. 2, The 18F multifunctional cystostomy trocar was rotated left and right slowly and stabbed into the abdominal cavity under ultrasound monitoring. The sharp-headed trocar core was replaced by the blunt-headed trocar core after breaking through the abdominal wall, and insertion continued in the direction of the vesicorectal fossa (or rectouterine fossa). The blunt-head trocar core was pulled out after reaching the target area. The guidewire and catheter are placed inserted into the pelvic cavity through the outer sheath of the trocar and move it to the Douglas fossa guided by ultrasound. Liquid entering the vesicorectal pouch (or rectouterine pouch) concomitant with catheter injection of normal saline was observed under ultrasound to confirm correct catheter tip placement.

#### 2.2.2. Open surgical placement of the PDC.

The position of the catheter deep cuff and exit site were marked before surgery. A PDC of appropriate length was selected according to the patient’s body type. Using a conventional disinfection towel, a longitudinal incision was made through the skin and subcutaneous tissue to expose the anterior sheath of the rectus abdominis under local infiltration anesthesia. The rectus abdominis was passively separated to expose the posterior sheath, and a small opening was cut to allow the PDC to pass through to the peritoneum. Following purse-string suture, the PDC end was moved into the cesicorectal fossa (or uterorectal fossa) through a small incision using a stainless steel guide wire. The proper placement was confirmed by linear saline outflow upon injection. The pouch was then ligated, and after ensuring that there was no leakage around the PDC, the inner polyester sleeve of the catheter was embedded into the anterior sheath of the rectus abdominis. The anterior sheath of the rectus abdominis was then sutured. A subcutaneous tunnel was established and externally connected via a titanium joint and a short tube. Finally, subcutaneous tissue and skin were sutured.

### 2.3. Postoperative management

Both patient groups received heparin saline to seal the catheter after surgery. On the second day after surgery, PD fluid (4 infusions of 500 mL each) was injected into the abdominal cavity to ensure that the catheter was unobstructed. The catheter was then fixed to the body surface, and patients were encouraged to get out of bed for activities in the early stage after surgery. The surgical dressing was changed once every three days until the stitches were removed. Routine maintenance of the catheter exit-site was conducted every day. At the same time, the patients were educated on PD home treatment, including early identification and treatment of peritonitis and catheter exit-site infection. Patients receiving color ultrasound-guided percutaneous PDC placement using the multifunctional bladder paracentesis trocar (Group A) started ambulatory continuous PD seven days after surgery, while patients receiving open surgical placement started ambulatory continuous PD 2 weeks after surgery.

Possible conditions requiring catheter removal include difficult drainage, failure of manual reduction due to omentum wrapping and displacement, refractory peritonitis, recurrent peritonitis, fungal peritonitis, refractory exit, and tunnel infection, mycobacterial peritonitis, and multiple intestinal bacterial infectious peritonitis. Catheters were reinserted following relief of peritonitis.

### 2.4. Clinical outcome assessment

Data recorded during catheterization included incision length, surgery time (from the beginning of local anesthesia to the end of catheterization), and intraoperative complications. Conditions monitored after catheterization included fluid drainage (obstructed or unobstructed) and the need for an analgesic pump after surgery. The day following surgery, abdominal plain film radiography was performed to confirm the catheter position. The fluid injection was then performed to confirm smooth abdominal fluid drainage and no turbidity of flushed PD fluid. Primary outcomes were clinical success rate of PDC within 2–4 weeks after catheterization, the occurrence of catheter-related mechanical complications (such as catheter displacement, leakage, hernia, and inadequate drainage), and infection (peritonitis, incision infection, tunnel and exit-site infection). Outpatient follow-up was conducted once a month, and the catheter survival rate was recorded at 12 months after surgery.

Clinical success was defined as smooth drainage within 2–4 weeks after catheterization without catheter tip displacement requiring surgical correction or other complications leading to catheter removal. Cases with catheter dysfunction, such as difficult liquid injection or inadequate drainage, were still defined as clinical successes if the problems could be rectified using conservative methods such as walking on tiptoe, hopping up stairs, or relaxing the bowels.

Catheter-related infections include peritonitis, tunnel infection, and exit-site infection. Criteria for diagnosis of peritonitis were as follows: (1) abdominal pain and turbid PD fluid with or without fever; (2) white blood cell count in PD effluent >100 × 10^6^/L, and proportion of neutrophils > 50%; (3) growth of pathogenic microorganisms from PD effluent in culture. The diagnosis was confirmed by at least two of these conditions. Cases with catheter-related infections after surgery were still considered successful if these signs were reversed by two weeks of antibiotic treatment, while cases requiring catheter removal or exhibiting infection recurrence were deemed clinical failures.

### 2.5. Statistical methods

SPSS17.0 software was used for all statistical analyses. categorical variables were compared between groups by chi-square test and continuous variables by independent samples *t* test. Kaplan–Meier analysis was used to evaluate PDC survival and group differences were assessed by log-rank test. Continuous variables are expressed as mean ± SD and categorical variables as counts and percentages. A *P* < .05 (two-tailed) was considered significant for all tests.

## 3. Results

The clinicodemographic features of the subjects are summarized in Table 1. Among these patients, 105 received ultrasound-guided percutaneous PDC placement using a multifunctional bladder paracentesis trocar (Group A), while 107 received open surgical placement (Group B). There were no significant differences in sex ratio, mean age, etiology, and history of previous abdominal surgery between groups (*P* < .05).

The surgical characteristics and early postoperative complications of the two groups are summarized in Table 2. Compared to open surgical placement, Ultrasound-guided trocar-assisted percutaneous placement required a smaller incision and shorter surgical time (*P* < .05). No patient in the percutaneous group required analgesics after catheterization. Abdominal plain film radiography revealed correct catheter location on the day following insertion in all patients of both groups, and there were no cases of major organ injury, severe abdominal hemorrhage, catheter displacement, peritubular leakage, incision hernia, peritonitis, exit infection, or tunnel infection. In the open surgery group, one patient suffered intraoperative hemorrhage, One additional patient suffered minor organ injury during surgery, and two required analgesia pumps whose cisual analog score (VAS) was 4–6 on the day of surgery. There was no significant difference in total intraoperative complications between groups (*P* > .05). Early complications were defined as those occurring within one month after catheterization. The combined incidence of early catheterization-related peritonitis, peritubular leakage, initial drainage obstruction, and catheterization failure was significantly lower in Group A (P < .05). The combined incidence of tunnel infection, exit-site infection, and catheter tip displacement was higher in the open surgical placement group, but the group difference did not reach statistical significance (*P* > .05).

**Table 2:** Operative characteristics and early* complications.

Variable	Group A (n = 105)	Group B (n = 107)	*P* value
Operation time (min)	20.76 ± 1.83	37.73 ± 2.83	.032
length of incision (cm)	2–4	4–6	–
Postoperative analgesic needs	0	2	.43
Bowel perforation	0	1	.65
Hemorrhage in rectus muscle or pelvic cavity	0	1	.43
Poor initial drainage [n (%)]	5 (4.76%)	11 (10.28%)	.046
peritonitis [n (%)]	0	6 (5.6%)	.02
Tunnel infection [n (%)]	0	0	–
Exit infection [n (%)]	0	2 (1.87%)	.26
Catheter migration [n (%)]	1 (0.95%)	5 (4.67%)	.59
Leakage	0	3 (2.80%)	.047
Mortality N (%)	0	0	–
Primary failure [n (%)]	4 (3.81%)	11 (10.28%)	.039
Total mortality	10 (9.52%)	38 (35.51%)	.014

The clinical success rate of PDC placement is shown in Table 3. The clinical success rate was higher in Group A than in Group B (96.19% vs. 89.91%, *P* < .05) but did not differ between group members with previous abdominal surgery history (20% vs.18.18%, *P* > .05). Potential causes of catheter failure include omentum packing, protein plugging, catheter displacement, and peritonitis. There were three total cases of failure requiring a surgical reduction in Group A, one case each of omentum packing, catheter displacement, and protein block. These rates did not differ from Group B. Alternatively, the rate of peritonitis requiring catheter removal was significantly lower in Group A (*P* < .05).

**Table 3:** Clinical success of peritoneal dialysis catheter insertion.

Variable	Group A (n = 105)	Group B (n = 107)	*P* value
Functioning catheter, N (%)	101 (96.19%)	96 (89.72%)	.046
Previous abdominal operation, N	5	9	–
Functioning catheter, N (%)	4 (80%)	7 (77.78%)	.11
Virgin abdomen, N	100	98	–
Functioning catheter, N (%)	97 (97%)	87 (88.78%)	.043

As shown in Table 4, the total number of patients with late complications or catheter dysfunction was significantly lower in Group A than in Group B (27.09% vs. 51.38%) and included a significantly lower rate of peritonitis (10.48% vs. 16.51%, *P* = .09) and a numerically lower rate of Catheter drainage dysfunction(4.76% vs.10.09%, *P* = .11) and Dialysate leakage (0 vs.1.83, *P* = .23). Total exit-site plus tunnel infection rate was also significantly lower in Group A than B (0% vs. 6.42%, *P* = .02), while there were no significant differences in all other complications such as henia (*P* > .05).

**Table 4:** Catheter-related long-term* complications.

Variable	Group A, N = 27	Group B, N = 55	*P* value
Catheter displacement, N (%)	9 (8.57%)	16 (14.95%)	.09
Peritonitis, N (%)	11 (10.48%)	18 (16.82%)	.31
Exit and tunnel infections, N (%)	0 (0%)	6 (5.61%)	.02
Dialysate leakage, N (%)	0 (0%)	2 (1.87%)	.23
Hernia, N (%)	2 (1.90%)	2 (1.87%)	.47
Catheter drainage dysfunction, N (%)	5 (4.76%)	11 (10.28%)	.11

The 1-year technical survival rate and reasons for removal/re-insertion are listed in Table 5. In Group A, 9 patients required catheter removal due to peritonitis (3 cases), catheter obstruction (1 case), omentum packing (3 cases), or catheter displacement (2 cases). All were re-catheterized after surgical reduction or catheter removal. In Group B, 21 cases required catheter removal due to peritonitis (7 cases), protein block (1 case), Omental wrapping (4 cases), Dialysate leakage(2 cases), or catheter displacement (7 cases).

**Table 5:** Factors associated with 1-year technical survival of catheters.

Variable	Group A, N = 9	Group B, N = 21	*P* value
Omental wrapping, N (%)	3 (2.86%)	4 (3.74%)	.39
Catheter displacement, N (%)	2 (1.90%)	7 (6.54%)	.12
Peritonitis, N (%)	3 (2.86%)	7 (6.54%)	.14
Dialysate leakage, N (%)	0	2 (1.87%)	.10
catheter obstruction, N (%)	1 (0.95%)	1(0.93%)	.52

**Figure 3.**
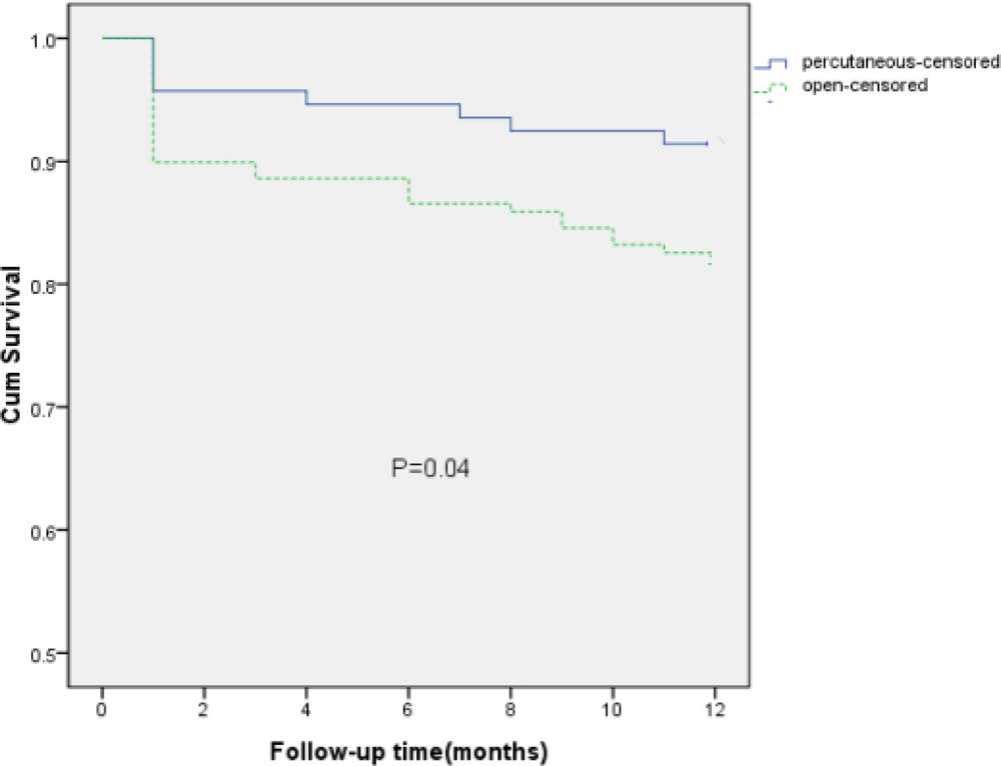
Kaplan–Meier analysis of catheter survival in the percutaneous using our technique (Group A)and open(Group B)groups (censored for catheter-related complications) (*P* = .04).

The 1-year PDC survival curves for Groups A and B are compared in Fig. 3. The 1-year survival rate was significantly higher in Group A compared to Group B (91.43% vs. 80.73%, *P* = .04 by log-rank test).

## 4. Discussion

Peritoneal dialysis has been used clinically since the 1950s,^[8]^ and is still a major treatment for patients with terminal-stage kidney disease. There are many catheterization methods in use for PD, such as open surgery, percutaneous placement, and laparoscopy, but improving the success rate of catheterization and reducing complications are still major challenges faced by nephrologists. In this study, we modified the blind Seldinger technique by incorporating ultrasound guidance and use of a multifunctional bladder paracentesis trocar for PDC placement and compared success and complication rates to conventional open surgical placement. Incidence rates of vascular injury during PDC placement have ranged from 0.02% to 0.5% and intestinal injury has averaged around 3% across previous studies.^[9–11]^ None of the patients in Group A suffered from intraoperative hemorrhage or organ injury, consistent with previous study results.^[12]^ In addition, the operative incision and surgical times were shorter in Group A than in Group B (both *P* < .05). This superior outcome is likely attributable to color ultrasound guidance, which allowed the operator to visualize the internal conditions of the abdominal cavity for greater placement accuracy in the cesicorectal fossa (or uterorectal fossa), while the percutaneous trocar kit provided integrated functions for percutaneous placement, expansion, and guidance. This combination permits the use of a smaller incision for catheterization, thereby reducing surgery time.

It is reported catheterization success rates of 77% using open surgery and 70% using laparoscopy,^[13]^ while Jwo et al.^[14]^ and Tsimoyiannis et al.^[15]^ reported success rates using laparoscopy of 84% and 100%, respectively. The overall clinical success rate of catheter placement was significantly higher in Group A than Group B, as was the success rate among subgroups without previous abdominal surgery, while success rates were equivalent among patients with previous abdominal surgery. Clinical failure in Group A was mainly due to omentum packing-associated catheter displacement and protein block. Early complications (within 1 month after catheterization) were also significantly lower in Group A, including incidence rates of catheterization-related peritonitis, peritubular leakage, and initial drainage obstruction. Similarly, the incidence rates of tunnel infection, exit-site infection, and catheter tip displacement were lower in Group A, although the differences did not reach statistical differences. In previous studies, peritoneal leakage and associated capsular leakage, hernia, genital swelling, or pleural effusion was the most common non-infectious complication, with an incidence rate of up to 19%.^[5,14,16]^ Hu et al.^[17]^ adopted a nephroscopic single-channel catheterization and catheter fixation method and found that reducing the number of abdominal incisions could decrease the incidence of peritoneal leakage. Zhu et al.^[18]^ and Takashi et al.^[19]^ reached similar conclusions. In the current study, Group A required less time to initiate PD after surgery compared to Group B, and no Group A patient exhibited catheter leakage, mainly because the smaller surgical incision caused less damage to the rectus abdominis muscle during catheterization and resulted in faster healing.

Peritonitis is the most common complication of catheter-related infection. In this study, the incidence rates of peritonitis were lower in Group A both initially and after one year. In both groups, incidence rates increased with time, in accord with a previous study,^[20]^ but remained lower in Group A. Catheter displacement is another common complication. Again, the incidence of catheter displacement was slightly lower in Group A both initially and after one year. The main reason for the superior success in Group A is that the PDC tip can be accurately placed into the pelvic floor under visualization. To reduce the occurrence of catheter displacement, many clinicians have fixed the PDC tip. For example, Wang et al.^[21]^ invented a device called “Wang’s forceps” for assistance in percutaneous PFC placement and fixation of the catheter tip to the abdominal wall, and no patient showed catheter displacement during the 6-month follow-up. A previous clinical study placed the PDC tip in the Douglas fossa and sutured it to the bladder, uterus, or pelvic sidewall; however, the surgery was difficult and there was a high incidence of complications.^[22]^ Alternatively, Shen et al.^[23]^ used laparoscopic suture hernia forceps insertion and fixation and reported no catheter displacement during follow-up. This surgery avoids intra-abdominal laparoscopic sutures and reduces operation difficulty. Therefore, by fixing the PDC tip, it is possible to further reduce the incidence rate of catheter displacement.

The survival duration of the catheter is the most important factor for successful long-term PD implementation. A randomized trial by van Laanen et al.^[13]^ reported a one-year survival rate of only 70% using laparotomy and 60% using laparoscopy, while 3 prospective randomized trials employing laparotomy reported survival rates of 67%–84%,^[21–23]^ markedly lower than in Group A. Thus, percutaneous PD catheter insertion using a multifunctional bladder paracentesis trocar and ultrasound guidance may facilitate more successful PD.

## 5. Conclusion

In conclusion, Ultrasound-guided percutaneous PDC placement using a multifunctional bladder paracentesis trocar reduces surgery time, incision size, time to PD initiation, and both intraoperative and postoperative complication rates compared to open surgical placement. Furthermore, one-year catheter survival is higher. This ultrasound-guided trocar-assisted operation is relatively simple and inexpensive, and thus more applicable in newly established or smaller PD centers.

However, this study has several limitations. First, the sample size was insufficient to assess differences in rarer complications. Second, this was a retrospective study so we could not directly examine methods for additional clinical efficacy and safety. Finally, This is a single-center study involving a relatively small patient group, so results may be influenced by selection bias. Further prospective randomized control trials are required for verification and additional technical improvements.

## Author contributions

Conceptualization: JianBIn Zhang.

Data curation: Zhen Li.

Formal analysis: Zheng Fang.

Funding acquisition: Zhen Li.

Investigation: Hongyun Ding.

Methodology: Hongyun Ding, Zheng Fang.

Project administration: Jianbin Zhang.

Resources: Jiye Zhang, Jie Liu.

Software: Yi Li.

Supervision: Zhen Li.

Validation: YunLu Yu, Jie Liu.

Visualization: Zheng Fang, Yi Li.

Writing – original draft: Zhen Li, Zheng Fang.

Writing – review & editing: JianBin Zhang, Zhen Li.

## Footnotes

* Within one month of peritoneal dialysis catheter insertion.

* Three month after peritoneal dialysis catheter insertion.

## References

[1] Crabtree JH Chow KM. Peritoneal dialysis catheter insertion. Semin Nephrol. 2017;37:17–29. 28153191 10.1016/j.semnephrol.2016.10.004

[2] Yip T Lui SL Lo WK. The choice of peritoneal dialysis catheter implantation technique by nephrologists. Int Nephrol. 2013;2013:940–1065. 10.1155/2013/940106 PMC3569939 23431443

[3] Yang PJ Lee CY Yeh CC. Mini-laparotomy implantation of peritoneal dialysis atheters: outcome and rescue. Perit Dial Int. 2010;30:513–8. 20190027 10.3747/pdi.2009.00033

[4] Schmidt SC Pohle C Langrehr JM. Laparoscopic-assisted placement of peri-toneal dialysis catheters:implantation technique and results. Laparoendosc Adv Surg Tech A. 2007;17:596–599. 10.1089/lap.2006.0162 17907970

[5] Medani S Hussein W Shantier M. Comparison of percutaneous and open surgical techniques for first-time peritoneal dialysis catheter placement in the unbreached peritoneum. Perit Dial Int. 2015;35:576–85. 25082842 10.3747/pdi.2013.00003 PMC4597991

[6] Park YS Min SI Kim DK. The outcomes of percutaneous versus open placement of peritoneal dialysis catheters. World J Surg. 2014;38:1058–64. 24305922 10.1007/s00268-013-2346-5

[7] Zhen L Hongyun D Xue L. Ultrasound-guided percutaneous peritoneal dialysis catheter insertion using multifunctional bladder paracentesis trocar: a modified percutaneous PD catheter placement technique. Dialysis in seminar. 2020;33:133–139. 10.1111/sdi.12862 PMC7187385 32160357

[8] Blagg CR. The early history of dialysis for chronic renal failure in the United State: a view from Seattle. Am J Kidney Dis. 2007;49:482–96. 17336711 10.1053/j.ajkd.2007.01.017

[9] Wakeen MJ Zimmerman SW Bidwell D. Viscus perforation in peritoneal dialysis patients: diagnosis and outcome. Perit Dial Int. 1994;14:371–7. 7827188

[10] Safety communications-laparoscopic trocar injuries: a report from a U.S. Food and Drug Administration (FDA) center for Devices and Radiological Health (CDRH) Systematic Technology Assessment of Medical Products (STAMP) Committee: FDA safety communication. Available at: http://www.fda.gov/MedicalDevices/Safety?Al ertsandNotices/ucm197339.htm./ [access date April 20, 2013].

[11] Ash SR. Chronic peritoneal dialysis catheters:procedures for placement, maintenance and removal. Semin Nephrol. 2002;22:221–36. 12012308 10.1053/snep.2002.31710

[12] Cruz C. Implantation techniques for peritoneal dialysis catheters. Perit Dial Int. 1996;16(suppl 1):S319–21. 8728215

[13] van Laanen JHH Cornelis T2 Mees BM. Randomized controlled trial comparing open versus laparoscopic placement of a peritoneal dialysis catheter and outcomes: the CAPD I trial. Perit Dial Int. 2018;38:104–12. 29386303 10.3747/pdi.2017.00023

[14] Jwo SC Chen KS Lee CC. Prospective randomized study for comparison of open surgery with laparoscopic-assisted placement of Tenckhoff peritoneal dialysis catheter—a single center experience and literature review. J Surg Res. 2010;159:489–96. 19482306 10.1016/j.jss.2008.09.008

[15] Tsimoyiannis EC Siakas P Glantzounis G. Laparoscopic placement of the Tenckhoff catheter for peritoneal dialysis. Surg Laparosc Endosc Percutan Tech. 2000;10:218–21. 10961749

[16] Schmidt SC Pohle C Langrehr JM. Laparoscopicassisted placement of peritoneal dialysis catheters: implantation technique and results. J Laparoendosc Adv Surg Tech A. 2007;17:596–9. 17907970 10.1089/lap.2006.0162

[17] Hu JC Chiu KY Wang SS. A Modified application of peritoneal dialysis catheter implantation: a revolution from the laparoscope-to the nephroscope-assisted surgery. J Endourol. 2018;32:502–508. 29630389 10.1089/end.2018.0071

[18] Zhu W Jiang C Zheng X. The placement of peritoneal dialysis catheters: a prospective randomized comparison of open surgery versus“Mini-Perc” technique. Int Urol Nephrol. 2015;47:377–82. 25395078 10.1007/s11255-014-0877-9

[19] Yoshida T Nakamoto T Yoshida K. Comparison of nephroscope-assisted “Pulling Thread” technique and conventional open placement of peritoneal dialysis catheters in patients with end-stage renal disease. Surgical Techniques in Urology. 2016;97:261–265. 10.1016/j.urology.2016.06.019 27397096

[20] Cox TC Blair LJ Huntington CR. Laparoscopic versus open peritoneal dialysis catheter placement[J]. Surg Endosc. 2016;30:899–905. 26092021 10.1007/s00464-015-4297-4

[21] Wang H Wang Y Zhu J. Wang’s forceps-assisted percutaneous insertion and fixation of peritoneal dialysis catheter. Artif Organs. 2018;42:728–35. 29602176 10.1111/aor.13121

[22] Frost JH Bagul A. A brief recap of tips and surgical manoeuvres to enhance optimal outcome of surgically placed peritoneal dialysis catheters. Int J Nephrol. 2012;2012:251584. 22888425 10.1155/2012/251584 PMC3408654

[23] Shen Q Jiang X Shen X. Modified laparoscopic placement of peritoneal dialysis catheter with intra-abdominal fixation. Int Urol Nephrol. 2017;49:1481–8. 28455661 10.1007/s11255-017-1593-z

